# Subcutaneous fat necrosis in neonates with hypoxic ischaemic encephalopathy registered in the Swiss National Asphyxia and Cooling Register

**DOI:** 10.1186/s12887-015-0395-7

**Published:** 2015-07-09

**Authors:** Beate Grass, Lisa Weibel, Cornelia Hagmann, Barbara Brotschi, on behalf of the National Asphyxia and Cooling Register Group

**Affiliations:** Department of Paediatric and Neonatal Intensive Care, University Children’s Hospital Zurich, Steinwiesstrasse 75, 8032 Zurich, Switzerland; Department of Paediatric Dermatology, University Children’s Hospital Zurich, Steinwiesstrasse 75, 8032 Zurich, Switzerland; Department of Dermatology, University Hospital Zurich, 8091 Zurich, Switzerland; Clinic ofNeonatology, University Hospital Zurich, Frauenklinikstrasse 10, 8091 Zurich, Switzerland

**Keywords:** Hypoxic ischaemic encephalopathy, Subcutaneous fat necrosis, Register, Hypothermia therapy

## Abstract

**Background:**

Neonates with hypoxic ischaemic encephalopathy (HIE) are routinely treated with therapeutic hypothermia (TH) for 72 h in order to improve neurological outcome. Subcutaneous fat necrosis (SCFN) is an adverse event occurring in neonates with HIE.

**Methods:**

We analyzed risk factors for SCFN regarding demographic factors, cooling methods and deviation from target temperature range during hypothermia therapy. Data of all neonates registered in the National Asphyxia and Cooling Register in Switzerland between 2011 and 2013 were analyzed.

**Results:**

2.8 % of all cooled neonates with HIE developed SCFN. Perinatal and neonatal characteristics did not differ between neonates with and without SCFN. Applied cooling methods did not correlate with the occurrence of SCFN. In neonates with SCFN 83.3 % of all noted temperatures were within the target temperature range versus 77.5 % in neonates without SCFN. Neonates with SCFN showed 3.6 % of all measured temperatures below target temperature range compared to 12.7 % in neonates without SCFN.

**Conclusion:**

Subcutaneous fat necrosis in the neonate with HIE undergoing TH is a potential adverse event that seems to occur independently from the whole-body cooling method applied and proportion of temperature measurements outside target temperature range. In this cohort, moderate overcooling associated with moderate hypothermia (33.0–34.0 °C) does not seem to be an independent risk factor for SCFN. There is no correlation between the severity of HIE and incidence of SCFN.

**Electronic supplementary material:**

The online version of this article (doi:10.1186/s12887-015-0395-7) contains supplementary material, which is available to authorized users.

## Background

Therapeutic Hypothermia (TH) improves survival and neurodevelopment in neonates with moderate to severe hypoxic ischaemic encephalopathy (HIE) [1–4]. No major complications are associated with therapeutic hypothermia [3, 5]. However, in recent years, several cases of neonates with HIE suffering from subcutaneous fat necrosis (SCFN) have been reported in cooled [1, 6] and non cooled neonates [7, 8]. Whether SCFN is due to the primary insult of hypoxia/ischaemia or to the exposure to cold temperature or a combination of both still needs to be evaluated. Recent data suggest that TH could be an additional risk factor for SCFN in neonates with HIE [1, 6] although SCFN is also described in the pre cooling era. SCFN is characterized by firm palpable nodules or plaques with or without erythema appearing within the first weeks of life. SCFN is rare but may result in complications such as hypoglycemia, hypertriglyceridemia, thrombocytopenia and sometimes life-threatening hypercalcemia. The pathogenesis of these complications is not fully understood. It is partly associated with the perinatal ischemia/hypoxemia itself and with the resolution of the skin lesions with accompanying endocrinological changes. SCFN is self-healing, sometimes with residua such as skin atrophy and scarring [7, 9, 10].

The aim of this study is (i) to compare perinatal and neonatal characteristics of neonates with and without SCFN registered in the Swiss National Asphyxia and Cooling Register, (ii) to identify risk factors for SCFN and (iii) to evaluate cooling management and different cooling methods with regard to the development of SCFN.

## Methods

A National Asphyxia and Cooling Register was introduced in Switzerland in 2011. All nine Swiss tertiary level neonatal intensive care units and two paediatric intensive care units are part of the register. The conduction of the register including data collection, data analysis and data publication has been approved by the federal committee of experts on professional secret in medical research (“Eidgenoessische Expertenkommission fuer das Berufsgeheimnis in der medizinischen Forschung”). There is no need for parental consent, because the register is anonymous. A questionnaire for each neonate with registered SCFN was sent retrospectively to the centers (Additional file 1).

Neonates with hypoxic ischaemic encephalopathy cooled or not cooled were registered. Newborn infants were cooled according to a register cooling protocol [5]. The register documents are available online (http://www.neonet.unibe.ch/php/manuel.php?locator=tabs4acc2). Neonates undergoing therapeutic hypothermia were cooled for 72 h with a target temperature range of 33.0–34.0 °C followed by a rewarming period (rewarming 0.2–0.5 °C per hour). All centers used whole-body cooling, but different cooling methods were applied. We grouped the different cooling methods into three categories: active cooling (with cooling device; Blanketrol III (Cincinnati Sub-Zero Products, Inc., Cincinnati, OH, USA) (*n* = 1 unit, *n* = 8 neonates), the CritiCoolTM (MTRE, Charter Kontrom, Milton Keynes, UK) (*n* = 3 units, *n* = 35 neonates), Allon system (MTRE Charter Kontrom, Milton Keynes, UK) (*n* = 1 unit, *n* = 1 neonate), Arctic sun (Bard Medical S.A., Oberrieden, Switzerland) (*n* = 1unit, *n* = 7 neonates), passive cooling (natural cooling, neonate undressed, no external heating, *n* = 4 units, *n* = 33 neonates) and passive cooling in combination with gel or ice packs (*n* = 5 units, *n* = 44 neonates) in addition to natural cooling. All neonates had their core temperature monitored with either rectal or oesophageal probes.

All investigations including blood sampling and neuromonitoring was according to our register protocol (http://www.neonet.unibe.ch/php/manuel.php?locator=tabs4acc2).

Daily clinical examination reveals the diagnosis of SCFN during hospitalisation. After discharge all neonates with hypoxic ischaemic encephalopathy were followed by a paediatrician at least one week after discharge, at the age of one, two and four months.

### Statistics

Data are presented as frequencies and medians with ranges. Significant differences of perinatal/neonatal characteristics and cooling data between the group with and the group without SCFN were assessed using Mann–Whitney test and Pearson’s chi-squared test as appropriate. Statistical significance was defined as a p-value of less than 0.05. Statistical analyses were performed using SPSS software (Statistical Package for the Social Sciences, Version 20; SSPS Inc., ChicagoIllinois, USA).

## Results

### Patients

**Fig. 1:**
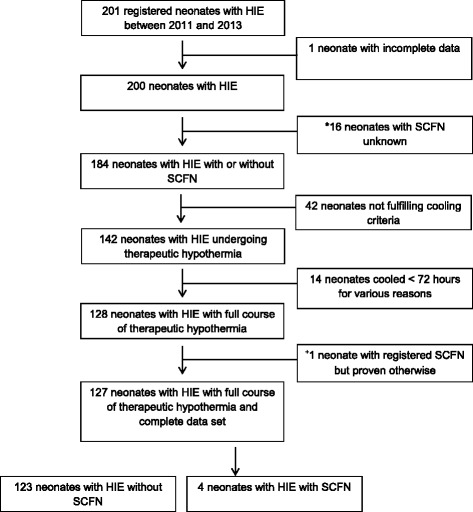
Flowchart patient recruitment. 201 neonates with HIE were registered between 2011 and 2013. Data of 17 neonates were incomplete and 42 neonates did not fulfill the cooling criteria. 142 neonates underwent therapeutic hypothermia, 128 of them underwent a full course (72 h hypothermia therapy followed by rewarming period). Four cooled neonates developed SCFN. *16 neonates with missing data concerning subcutaneous fat necrosis (SCFN). ^+^one neonate was registered with SCFN, but in the notes no SCFN was mentioned. HIE hypoxic ischaemic encephalopathy SCFN subcutaneous fat necrosis

201 neonates with HIE (cooled and non-cooled) were registered between 2011 and 2013. Patient recruitment is depicted in Fig. 1. Fourteen neonates died, none of them developed subcutaneous fat necrosis before death. All neonates who were not cooled had a mild HIE (defined by Sarnat stage 1 or Thompson stage <7) [11, 12], but did not meet cooling criteria. None exceeded the time window for initiation of therapy.

The incidence of subcutaneous fat necrosis in our study population was 2.8 % (4/142) of all cooled neonates and 3.2 % (4/127) of neonates with a full course of therapeutic hypothermia (TH for 72 h). No SCFN occurred or was reported in non-cooled neonates and in neonates cooled for less than 72 h. Two cooled neonates with SCFN were treated in the same center (incidence of SCFN for this center is 2/29 neonates (6.9 %)) whereas the remaining two cooled neonates with SCFN were treated in different centers (incidence of SCFN per center is 1/16 neonates (6.3 %) and 1/12 neonates (8.3 %), respectively). No neonate developed SCFN in the other 7 centers.

There were no statistically significant differences between the two groups (with and without SCFN) regarding perinatal and neonatal characteristics including gestational age, Sarnat and Thompson Scores (Table 1) [11, 12].

**Table 1:** Perinatal and neonatal characteristics of all cooled neonates with HIE (*n* = 142)

	With SCFN (*n* = 4)	Without SCFN (*n* = 138)	*p*-value
Gestational age [days]	278 [268–301]	279 [244–302]	ns
Birth weight [grams]	3340 [3190–3920]	3200 [1790–4700]	ns
Head circumference [cm]	34.75 [34.0–35.5]	34.50 [30.5–40.0]	ns
APGAR score 5 min	4 [1–7]	3 [0–10]	ns
APGAR score 10 min	7 [4–8]	5 [0–10]	ns
Umbilical artery pH	6.96 [6.90–7.05]	6.92 [6.54–7.43]	ns
Worst pH^a^	7.02 [6.90–7.19]	6.88 [6.50–7.46]	ns
Worst lactate[mmol/l] [mmol/l]^a^	18 [15.0–19.0]	15 [1.80–28.0]	ns
Sarnat Score on admission	1.5 [1.0–2.0]	2.0 [1.0–3.0]	ns
Thompson Score on admission	4.5 [3.0–6.0]	9.0 [2.0–15.0]	ns
Sarnat Score after TH	1.0 [1.0–1.0]	1.0 [1.0–3.0]	ns
Thompson Score after TH	1.0 [1.0–1.0]	3.0 [1.0–17.0]	0.05

All values given as median and range [in parenthesis]

*SCFN* subcutaneous fat necrosis, *TH* therapeutic hypothermia, *ns* not significant

^a^worst pH and worst lactate within first 60 min of life

The four neonates developing SCFN (two inborns, two outborns) were born after an uneventful pregnancy. None of the mothers had known maternal risk factors for the occurrence of SCFN such as diabetes mellitus/gestational diabetes, hypertension, preeclampsia, seizures, thyroid dysfunction or illicit drug consumption [7, 8, 10]. None of the neonates had known risk factors such as meconium aspiration or macrosomia [7, 8, 10]. Demographic details of the four neonates with SCFN are shown in Table 2.

**Table 2:** Characteristics of all 4 neonates with SCFN

SCFN	Case 1	Case 2	Case 3	Case 4
Sex	Female	Female	Male	Female
Gestationalage[weeks]	38 2/7	40 2/7	39 1/7	40 0/7
Birth weight [grams]	3920	3480	3400	3190
Mode of delivery	Emergency caesarian section	Instrumental delivery	Instrumental delivery	Emergency caesarian section
Age at appearance of SCFN [days]	16	3	6	4
Cooling method applied	Passive plus ice packs	Passive plus ice packs	Active (Criticool System)	Active (Criticool System)
Localization of SCFN	Left scapula region and dorsal upper arm	Back	Back	Upper back
Appearance of SCFN	Red nodules, plaques	Red nodules, plaques	Palpablenodules, plaques	Red nodules, plaques
Diagnosis by	Neonatologist	Neonatologist	Neonatologist	Neonatologist
Therapy	Conservative	Conservative	Conservative	Hydration, low calcium formula, no vitamin D supplementation, analgesia
Thrombocytopenia	No	No	No	No
Hypoglycemia	No	No	No	No
Calcium level ionized (total) [mmol/l]	1.41 (2.49)	1.33	1.5	1.54 (2.77)
Follow-up	Yes	Yes	Yes	Yes
Who	Pediatrician, pediatric surgeon	Pediatrician	Pediatrician	Pediatrician, endocrinologist
Examination	Clinical follow-up	Clinical follow-up and calcium level	Clinical follow-up	Clinical follow-up, calcium level and ultrasound of kidneys

*SCFN* subcutaneous fat necrosis calcium level: routinely ionized calcium is measured

### Cooling

Those neonates who were cooled less than 72 h were excluded from further analysis. All included neonates were cooled for 72 h and developed no severe complications of TH including severe coagulopathy, thrombocytopenia, pulmonary hypertension or infection.

**Fig. 2:**
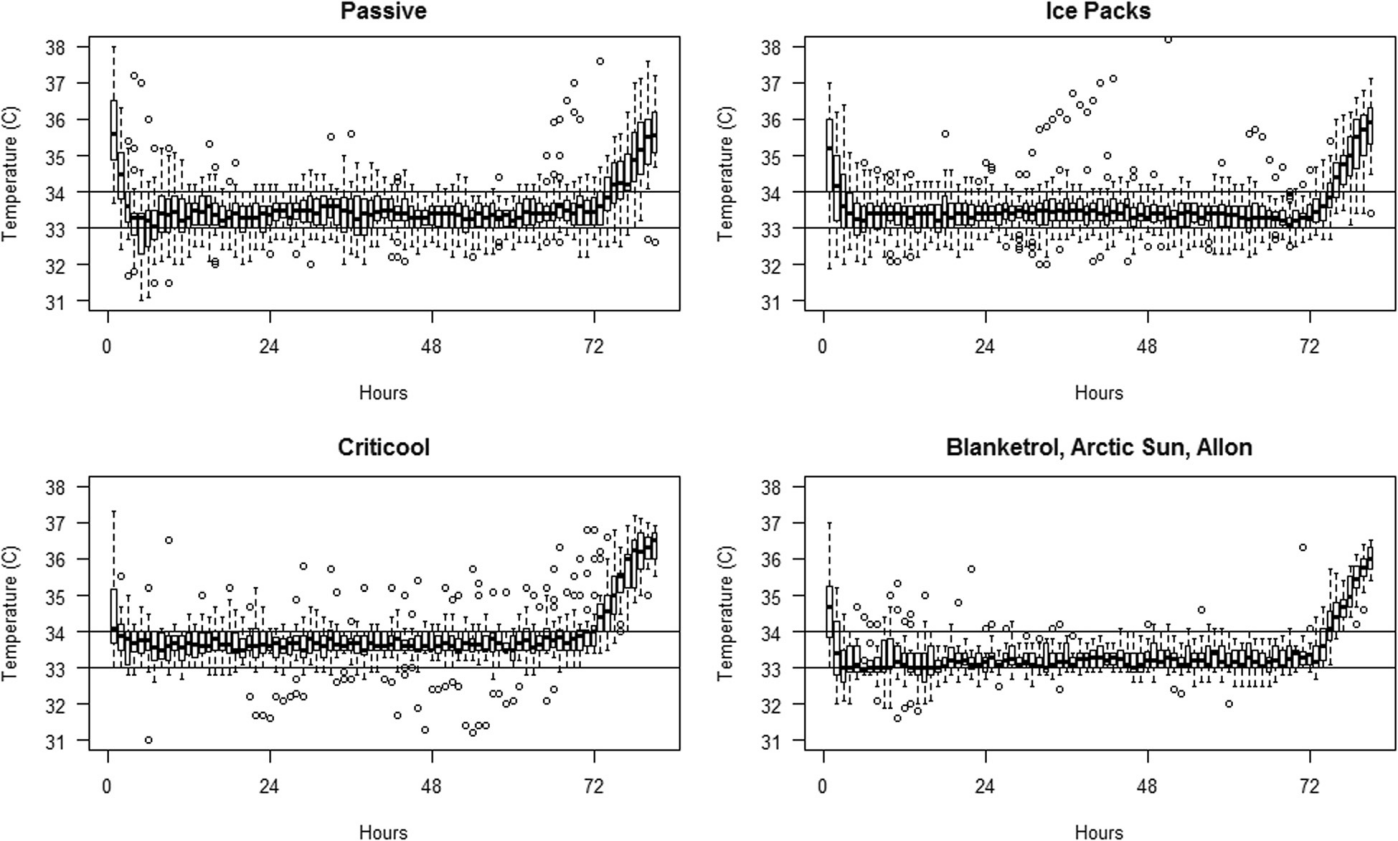
Boxplots of body temperature of cooled neonates over time. Passive cooling (*n* = 33), passive cooling with gel/ice packs (*n* = 44), Criticool system (*n* = 35), Blanketrol III, Arctic Sun and Allon system (*n* = 15)

50/127neonates (39.4 %) were actively cooled, 33/127 neonates (26.0 %) passively, and 44/127 neonates (34.6 %) passively in combination with gel/ice packs. In all neonates undergoing TH temperature was monitored every hour. The cooling methods did not significantly influence the percentage of temperature measurements in target temperature range (passive cooling 73.3 %, passive with additional ice/gel packs 79.5 %, active 78.9 %, *p* = 0.35), Fig. 2).

The incidence of SCFN with passive cooling in combination with gel/ice packs is 4.5 % (2/44 neonates), with active cooling 4 % (2/50 neonates) and no SCFN was noted in the neonates passively cooled. Data shown suggests that active cooling and application of ice/gel packs could be a risk factor for SCFN, but SCFN occurred independently from the cooling method (Pearson chi-squared test *p* = 0.223). Seventy percent of all actively cooled neonates were treated with the Criticool system, therefore it might not be surprising that the two neonates which had SCFN in the actively cooled group were treated with the Criticool system.

Of all noted temperatures 83.3 % were within the target temperature range (33.0–34.0 °C) in neonates with SCFN versus 77.5 % in neonates without SCFN. Thirteen percent of the temperature values were above 34.0 °C in the group with SCFN versus 9.7 % in neonates without SCFN. Neonates with SCFN showed 3.6 % of all measured temperatures below the target temperature range compared to 12.7 % in neonates without SCFN. SCFN occurred independently of the percentage of measured temperatures within target temperature range (within/below/above, Pearson chi-squared test *p* = 0.199)

## Discussion

In our study population the incidence of SCFN in cooled neonates with HIE was 2.8 % which is consistent with previous reports [6, 9]. Overlooking the past forty years, approximately 20 articles (mostly case reports) have been published on SCFN occurring in context with HIE in neonates (Additional file 2: Table S1). Most of the studies investigated non cooled neonates with HIE and SCFN.

Certain maternal risk factors such as maternal diabetes mellitus, hypertension, preeclampsia, seizures, thyroid dysfunction or illicit drug consumption have been mentioned in the literature [7, 8, 10]. None of them were found in our study population nor could we find the known neonatal risk factors such as meconium aspiration, hypoglycemia or macrosomia [7, 8, 10]. In our study none of the analyzed perinatal and neonatal characteristics showed statistical significance between neonates with and without SCFN (Table 1). We may have been unable to confirm the risk factors described by Mahé et al. and Burden et al., as they analyzed non-cooled neonates in contrast to our neonates who underwent TH [7, 8]. Additionally our sample size might be too small to detect an association of the known risk factors.

In line with the proposed pathophysiology of SCFN one might expect SCFN to occur in neonates with more severe HIE independently of cooling. However, in our study population there was no significant difference of the Sarnat and Thompson Score, Apgar Score or metabolic parameters within the first hour of life between both groups [11, 12].

Certain body areas with bony protuberances are most susceptible to SCFN [10, 13], namely the back and the occipital scalp as seen on Additional file 3: Figure S1 (parental consent was obtained to publish this picture). This is also true in our collective and the question arises whether the occurrence of SCFN correlates to the cooling technique applied. However, our data does not show any association between applied cooling method and the incidence of SCFN. It seems to be a coincidence that no SCFN occurred in the group of passively cooled neonates with HIE.

Nevertheless, as in our study population most neonates with HIE are bedded in supine position with SCFN occurring on the back, regular changing of position is advocated to reduce any damage. Regular mobilization should be routinely integrated in the nursing protocol, if the neonate is stable enough [9, 14].

Our findings support the data of the TOBY Register that moderate hypothermia during 72 h could be an additional risk factor for SCFN, although our sample size is quite small and we investigated only 42 non cooled neonates as a control group [6]. This does not mean that SCFN does not appear in non-cooled neonates with HIE, because birth asphyxia is a risk factor per se.

Of interest, SCFN occurred independently of the percentage of measured temperatures within the target temperature range during TH. Compared to the findings that severe hypothermia (core temperature below 28.0 °C) is a described risk factor for developing SCFN [15], overcooling below 33.0 °C (but above 31.5 °C) did not emerge as a risk factor in our study population.

SCFN can still occur after discharge from hospital. This is one of the reasons why SCFN might be well unrecognized and thus undiagnosed. Therefore, it is important to inform the parents and the outpatient paediatricians of the nature of SCFN.

### Limitations

As we analyzed the data of the National Asphyxia and Cooling Register we describe the incidence of SCFN during hospitalization. After discharge all neonates were regularly followed by an outpatient paediatrician one week after discharge, at the age of one, two and four months. However they are not followed by the initial care takers.

Numbers of SCFN in our study population are small and conclusions are difficult to draw. Firm conclusions cannot be supported by this data, but they show a tendency.

Furthermore the SCFN were evaluated with a retrospective questionnaire which entails certain limitations. In the future as soon as SCFN is diagnosed a real time questionnaire needs to be obtained. A national register therefore permits to follow a trend and is important to discover adverse events and to improve patient management.

## Conclusion

Subcutaneous fat necrosis of neonates with HIE undergoing TH is an adverse event that seems to occur independently from the severity of HIE, the cooling method applied and the proportion of measured temperatures outside target temperature range. In this cohort moderate overcooling associated with moderate hypothermia (33–34 °C) does not seem to be a risk factor for SCFN.

## Additional files

Additional file 1:
**Questionnaire for subcutaneous fat necrosis (SCFN).**
Additional file 2: Table S1. Published studies of SCFN in neonates with HIE [16–23]. Additional file 3:
**Neonate with HIE and SCFN.**

## Abbreviations

SCFN: Subcutaneous fat necrosis
TH: Therapeutic hypothermia
HIE: Hypoxic ischaemic encephalopathy
Ns: Not significant
