# Supplementary material for: Subcutaneous fat necrosis in neonates with hypoxic ischaemic encephalopathy registered in the Swiss National Asphyxia and Cooling Register
Source: BMC Pediatr. 2015 Jul 9;15:73. doi: 10.1186/s12887-015-0395-7 (PMC4496817; doi:10.1186/s12887-015-0395-7)
Supplement: Additional file 1: — Questionnaire for subcutaneous fat necrosis (SCFN). [file 12887_2015_395_MOESM1_ESM.docx]

**Questionnaire for subcutaneous fat necrosis (SCFN)**

**Birth date**

**sex**

**gestational age**

**Patient age at presentation of the subcutanoeous fat necrosis**

**Localisation of the SCFN**

**Diagnosis made by**

**dermatologist**

**neonatologist**

**biopsy**

**Therapy of SCFN ?**

**Was the calcium level measured ?**

**What was the maximum calcium level ?**

**Follow up of the SCFN ?**

**If yes, follow up by pediatrician, neonatologist or dermatologist ?**

**What was examined during the follow up ?**

- **Clinical examination ?**
- **Calcium level ?**
