# Supplementary material for: Subcutaneous fat necrosis in neonates with hypoxic ischaemic encephalopathy registered in the Swiss National Asphyxia and Cooling Register
Source: BMC Pediatr. 2015 Jul 9;15:73. doi: 10.1186/s12887-015-0395-7 (PMC4496817; doi:10.1186/s12887-015-0395-7)
Supplement: Additional file 2: Table S1. — Published studies of SCFN in neonates with HIE [16–23]. [file 12887_2015_395_MOESM2_ESM.docx]

| **Table S1:** Published studies of SCFN in neonates with HIE  ***author** | **study design** | **cooling method** | **SCFN**  **localisation** | **SCFN**  **age at appearance [DOL]** | **SCFN**  **diagnostics** | **SCFN**  **Calcium level / complications** | **SCFN**  **therapy** |
| --- | --- | --- | --- | --- | --- | --- | --- |
| **Kuboi**  **2013 (15)** | case report and discussion | active  (Medi-Cool head cooling) | cheeks and back | 24 | clinical | calcium normal; no complications | no specific therapy |
| **Calisici**  **2013 (13)** | case report and discussion | passive | back and dorsal arm | 3 | punch biopsy | calcium 6.8 mg/dl; hypoglycemia and polycythemia | glucose infusion, partial blood exchange, hydration |
| **Woods**  **2012 (16)** | case report and discussion | active  (induced cooling) | lumbar region | 5 | clinical | calcium level unknown; hypoglycemia | glucose infusion; analgetics |
| **Filippi**  **2012 (8)** | retrospective study  (2 cases) | active  (Blanketrol) | case 1: neck lateral, back  case 2: back, dorsal thighs, armpits | case 1: 23  case 2: 10 | clinical | case 1: calcium normal, no complications  case 2: calcium 8.3 mg/dl; thrombocytopenia | no specific therapy |
| **Hogeling 2012 (12)** | case reports (3 cases) and literature review | active  (Blanketrol) | case 1: occipital scalp, neck, back  case 2: nape until lower back, arm  case 3: both arms dorsal, back | case 1: 9  case 2: 11  case 3: 5 | case 1: clinical and biopsy  case 2: biopsy  case 3: biopsy | case 1: calcium normal; no complications  case 2: calcium 11.5 mg/dl, thrombocytopenia  case 3: no data available | No specific therapy |
| **Strohm**  **2011 (5)** | retrospective analysis (12 cases) discussion | active  (cooling mattress) | buttocks, back, arms | median 6  (range 4-42) | clinical | median calcium 2.98 mmol/l  (range 1.5 – 5.1 mmol/l); one case with calcium 3.37 mmol/l and nephrocalcinosis | low calcium formula, diuretics |
| **Oza**  **2010 (9)** | case report and comment | active  (cooling blanket) | upper back, buttocks, dorsal arms | 6 | clinical and biopsy | Calcium 11.2 mg/dl; no complications | no specific therapy |
| **Zifman**  **2010 (17)** | case report and discussion | cooling procedure not specified | occipital scalp, cheeks, arms, back | 7 | clinical | calcium 13.7 mg/dl; parathyroid hormone suppressed, 1,25-dihydroxyvitaminD_3_ elevated, nephrocalcinosis | hydration, steroids |
| **Gherghereh-chi**  **2008 (18)** | case report and discussion | no cooling | back | 22 | clinical | calcium 15.6 mg/dl; hypoglycemia, thrombocytopenia, parathyroid hormone suppressed, 1,25-dihydroxyvitaminD_3_ elevated, nephrocalcinosis,  brain calcifications | hydration, steroids, diuretics |
| **Mahé**  **2007 (6)** | retrospective study  (16 cases) | no cooling | trunk, back, cervical, head, arms, legs | median 4  (range 2 – 9) | clinical (9 cases) and biopsy (7 cases) | calcium median 3.3 mmol/l (range 2.8 – 4.8 mmol/l); nephrocalcinosis (3 cases); hypertriglyceridemia (1 case) | low calcium and vitamin D formula, analgesia, diuretics, steroids, bisphos-phonate |
| **Aucharaz**  **2007 (19)** | Case report and discussion | no cooling | back, groin, thighs | 4 | clinical | calcium 3.99 mmol/l; nephrocalcinosis | hydration, diuretics, steroids |
| **Tran**  **2003 (20)** | case report and discussion | no cooling | back, buttocks | 3 | clinical | calcium 18.4 mg/dl; thrombocytopenia, hypoglycemia, parathyroid hormone suppressed, 1,25-dihydroxyvitaminD_3_ elevated, nephrolithiasis, microcalcification | hydration, low calcium formula, diuretics, polycitrate, surgery |
| **Wiadrowski 2001 (21)** | case report and discussion | passive plus ice packs | thighs, back, buttocks | 2 | clinical and biopsy | calcium 3.74 mmol/l; no complications | hydration, low calcium and vitamine D formula, analgesia,  steroids, diuretics, bisphos-phonates |
| **Burden**  **1999 (7)** | retrospective study (11 cases) and discussion | no cooling | trunk, thighs, arms, cheeks, buttocks, back | range 2 -30 | clinical and biopsy (5 cases) | calcium elevated (4 cases, maximum calcium 4.9 mmol/l); hypoglycemia (4 cases), thrombocytopenia (5 cases), hypertriglyceridemia (1 case) | hydration,  diuretics, steroids,  calcitonin |
| **Duhn**  **1968 (22)** | case report (2 cases) and discussion | no cooling | case 1: lower back  case 2: trunk, arms, legs | case 1: 14  case 2: 35 | case 1: clinical  case 2: biopsy | case 1: calcium 9.7 mg/dl; calcification  (X-ray)  case 2: calcium 9.8 mg/dl; calcification  (X-ray) | no specific therapy |

*author, year of publication, reference number in parenthesis

DOL day of life; SCFN subcutaneous fat necrosis
