# Supplementary figures and images for: Subcutaneous fat necrosis in neonates with hypoxic ischaemic encephalopathy registered in the Swiss National Asphyxia and Cooling Register
Source: BMC Pediatr. 2015 Jul 9;15:73. doi: 10.1186/s12887-015-0395-7 (PMC4496817; doi:10.1186/s12887-015-0395-7)

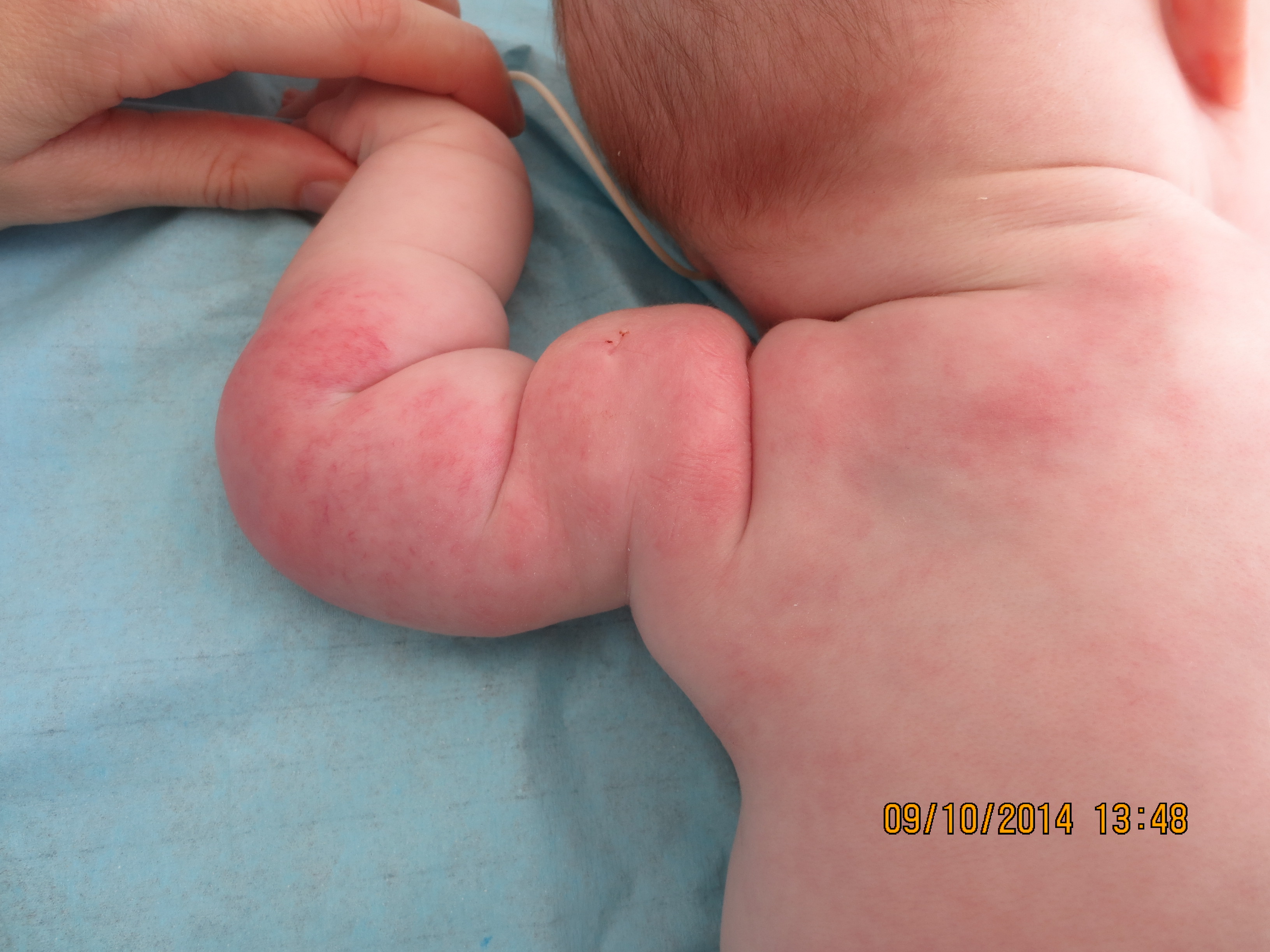

Supplement: Additional file 3: — Neonate with HIE and SCFN. [file 12887_2015_395_MOESM3_ESM.jpeg]
